# A historical perspective on malaria control in Brazil

**DOI:** 10.1590/0074-02760150041

**Published:** 2015-09

**Authors:** Sean Michael Griffing, Pedro Luiz Tauil, Venkatachalam Udhayakumar, Luciana Silva-Flannery

**Affiliations:** 1 Centers for Disease Control and Prevention, Center for Global Health, Division of Parasitic Diseases and Malaria, Malaria Branch, Atlanta, GA, USA; 2 Atlanta Research and Education Foundation, Atlanta, GA, USA; 3 Universidade de Brasília, Centro de Medicina Tropical, Brasília, DF, Brasil

**Keywords:** Brazil, malaria, Plasmodium, vivax, falciparum, drug resistance, control, history

## Abstract

Malaria has always been an important public health problem in Brazil. The early history of Brazilian malaria and its control was powered by colonisation by Europeans and the forced relocation of Africans as slaves. Internal migration brought malaria to many regions in Brazil where, given suitable*Anopheles* mosquito vectors, it thrived. Almost from the start, officials recognised the problem malaria presented to economic development, but early control efforts were hampered by still developing public health control and ignorance of the underlying biology and ecology of malaria. Multiple regional and national malaria control efforts have been attempted with varying success. At present, the Amazon Basin accounts for 99% of Brazil’s reported malaria cases with regional increases in incidence often associated with large scale public works or migration. Here, we provide an exhaustive summary of primary literature in English, Spanish and Portuguese regarding Brazilian malaria control. Our goal was not to interpret the history of Brazilian malaria control from a particular political or theoretical perspective, but rather to provide a straightforward, chronological narrative of the events that have transpired in Brazil over the past 200 years and identify common themes.

The early history of Brazilian malaria and its control was powered by European colonisation and the forced relocation of Africans as slaves. These immigrants brought malaria to many regions in Brazil where, given suitable anopheline mosquito vectors, it could thrive. Almost from the start, officials recognised the problem malaria presented to economic development, but control efforts were hampered by limited public health control and ignorance of the underlying biology and ecology of malaria. As World War II ended, Brazil was poised to confront this national scourge. It did so through multiple regional and national efforts, each with varying success.

Here, we describe the grand sweep of Brazilian malaria and its control from the 1500s to the present. Our goal was not to interpret the history of Brazilian malaria control from a particular political or theoretical perspective, but rather to provide a straightforward, chronological narrative of the events that have transpired in Brazil over the past few hundred years and identify common themes. We view this review as a stepping off point for scholars interested in specific aspects of Brazilian malaria.

## The early history of Brazilian malaria and regional control - Railroads and rubber

Brazilian malaria was first reported as “tertian and quartan fevers” affecting the Tupinambá Indians in 1587 (Deane 1986, Coura et al. 2006). Molecular analysis suggests that *Plasmodium falciparum* malaria was introduced to Brazil with the African slave trade, potentially as early as 1560 (Yalcindag et al. 2012). There is more debate regarding the origin of *Plasmodium vivax* malaria in Brazil, but molecular data suggests it was either introduced once to South America 15,000-30,000 years ago or multiple times from a now extinct European *P. vivax* population less than 500 years ago (Taylor et al. 2013). Large scale epidemics were not reported during the colonial period (Deane 1986, Coura et al. 2006). However, the Baixada Fluminense, a lowland convergence of four river basins in the state of Rio de Janeiro (RJ), was filled with malarious swamps (Gadelha 1998).

After the colonial period, the largest drivers of malaria control intervention were the rubber industry and railroad construction (Deane 1986). Natural rubber was crucial to Brazil’s economy beginning in the middle of the XIX century (Stepan 2003). By the 1870s, migrants were fleeing northeastern droughts to seek work in the Amazon rubber industry. As immunologically naïve hosts, they were susceptible to malaria (Deane 1986, Coura et al. 2006). Simultaneously, efforts were made to interconnect the vast untapped lands of the Brazilian interior by trains and telegraph wires.

The abolition of slavery in 1888 disrupted the agricultural industry and the resultant disruption of irrigation and drainage systems at abandoned plantations led to the Baixada Fluminense, coastal plains of RJ and parts of the state of São Paulo (SP) being highly malarious for decades (Deane 1986). Anti-malaria commissions were started in 1891, but performed poorly due to limited funds, sporadic activity, ignorance and scattered administration (Gadelha 1998). In particular, Manaus, in the state of Amazonas (AM), went through four sanitary commissions for the control of tropical diseases between 1897-1913 (Schweickardt & Lima 2010). From 1890-1900, approximately 500,000 Brazilians migrated to the Amazon in a “chaotic flood,” with many becoming rubber tappers (Stepan 2003). From 1892-1906, 26.6 people per 10,000 died of malaria in Northeast Brazil, with approximately half originating from outside the region (Packard & Gadelha 1997). Malaria was present throughout Brazil by 1900. Each year, there were six million cases, which represented 50% of Brazil’s population (Coura et al. 2006).

The leading malaria institutes in Brazil were the Bacteriological Institute of São Paulo and the Domingos Freire Bacteriological Institute, founded in 1892 and 1893, respectively. Adolpho Lutz ran the Bacteriological Institute of São Paulo within a year of its founding. He created the first map of malaria in SP (Benchimol & Silva 2008).

Oswaldo Gonçalvez Cruz became the technical director of the Federal Serum Therapy Institute in Manguinhos, RJ in 1900 [later renamed the Oswaldo Cruz Institute (IOC)] (Deane 1988). His early published worked focused on malaria that occurred around RJ and a location in Baixada Fluminense near a railroad. Railroads were the driving force for most of the malaria interventions conducted by the Institute. In 1902, Oswaldo Cruz became the head director and Carlos Chagas joined the Institute (Benchimol & Silva 2008). Carlos Chagas would go on to publish papers on malaria prophylaxis which culminated in the proposal that malaria was an infection of the home (Deane 1988, 19, 1992). Arthur Neiva would later join the Institute in 1906, as did Adolpho Lutz (Stepan 2003, Benchimol & Silva 2008).

In 1903, Adolpho Lutz identified *Anopheles cruzi*, a mosquito that bred in bromeliads, as the vector spreading malaria along the Santos-São Paulo railroad (Lutz 1903, Dourado 1992, Benchimol & Silva 2008). He was led to this hypothesis by the observation that much of the railroad was built at 900 m, where stagnant water was uncommon. Adolpho Lutz was also the first to hypothesise that this was the cause of endemic malaria found in southern state of Santa Catarina (SC), where it had been reported since 1820. Unfortunately, foreign opposition to his hypothesis would limit control of this vector until the 1940s (Gadelha 1994).

While knowledge of the basic biology of malaria had recently grown, the strategies to confront it were still inchoate. From 1905-1906, Carlos Chagas was assigned to Itatinga, where malaria had halted the construction of a railroad. He initially surveyed anopheline species, their larvae habitats and their distance to domiciles. He identified elderly and young individuals that could act as parasite reservoirs. The local population lived in two large housing units without malaria protection and initial analysis suggested 30% were infected with malaria, typically *P. vivax* (Benchimol & Silva 2008).

Carlos Chagas applied “offensive” prevention methods and “defensive” methods inspired by Ronald Ross. Offensively, he killed adult mosquitoes within homes and nearby larvae. Defensively, he gave 50 cg quinine to workers every three days at the afternoon meal, which they accepted readily. He also destroyed larvae breeding sites near the two housing units, treated children and the chronically ill and isolated patients with active blood infection. Individual protection of workers was deemed impossible (Benchimol & Silva 2008).

Workers wore thin clothing that mosquitoes could bite through and it was too hot to wear thicker fabric, therefore gloves and face netting were useless. While such protection might work for “white collar” workers, it would not for manual labourers. Instead, labourers were confined to screened housing that only had one entrance through two screen doors in an airlock configuration. Yet even this method had to go through a trial by error stage, as the initial mesh used to screen housing was too large to keep out smaller vectors. Asking healthy workers to confine themselves to the sheds between dusk and after dawn was not considered acceptable, though by 1907 he proposed that workers could be confined for 1 or 2 h at dusk. Cases died off and there were none when he left in March, 1906 (Benchimol & Silva 2008).

In 1907, Carlos Chagas and Arthur Neiva travelled to the Baixada Fluminense, 60 km from the city of Rio de Janeiro, though Carlos Chagas would leave after only three months. There, workers were building waterworks and a central railroad (Agudelo 1990, Benchimol & Silva 2008). They were charged with providing malaria prophylaxis for the 3,500 workers damming the Xerém River (Stepan 2003). Work on the railroad in Xerém and Mantiqueira had halted due to epidemics that left 96% of workers infected with malaria (Agudelo 1990). A hospital was built at the end of the railroad and larval habitat was filled or rechanneled, including the destruction of bromeliads. Insecticides were applied to water deposits near domiciles. Larvae-eating fish were used and wells were coated with petroleum. All infected patients in the area were treated, regardless of whether they were involved in the public work projects and those with gametes were isolated (Benchimol & Silva 2008).

All workers were to take quinine every week or be dismissed, though Arthur Neiva noted that the dosage required to treat malaria increased over 20 months from 50 cg every three days to the same dose daily. The mandatory use of quinine was extremely unpopular with labourers. A worker who was fired for not taking his medication attacked the staff member responsible for administering quinine the next day and was killed. Another quinine administrator was harassed by an engineer and 100 labourers. After Arthur Neiva stated they should all be fired, this group visited the hospital (Benchimol & Silva 2008).

After three months, Chagas was called away to lead malaria interventions in the state of Minas Gerais (MG), near the Bicudo River. Almost all of the 1,500 workers in the area had been infected with malaria. The region was crisscrossed with bogs, bromeliads, rivers, streams and swamps where larvae could breed year round, leaving water sanitation futile. The constant movement of workers down the railroad line meant that personal and collective preventive measures were also ineffective. After symptoms disappeared, workers assumed they were cured, but instead they became chronically ill (Benchimol & Silva 2008).

In response, the medical commission established three measures that all personnel were to follow. One, all sick went to infirmaries and only left when authorised. Two, those considered epidemiologically dangerous, were isolated in covered wagons or sheds from dusk and only left at dawn. Three, all personnel took 50 cg of quinine every three days (though this seems to have changed to every other day and later every day). Those that did not comply were fined or dismissed. Carlos Chagas divided the population into the uninfected and infected. However, there were so many chronic cases that only the most serious were isolated. Early reports suggest that labourers argued that quinine was no longer needed when they appeared in good health and that the medication had side effects. Over time, this methodology reduced the number of relapses (Benchimol & Silva 2008).

In 1908, Arthur Neiva was hired to conduct an antimalaria campaign along a railroad that was attempting to link Bauru, SP, to Cuiabá, in the state of Mato Grosso (MT). Again, Arthur Neiva applied quinine as his chief intervention, though his experiences in the Baixada Fluminense convinced him to not make it mandatory. Instead, he unsuccessfully used advertising. His campaign focused more on alleviating symptoms than prevention with Neiva’s only commitment being that deaths from malaria should not increase. The railway made it to the banks of the Paraná River by 1910, but health was so poor that a bridge over the river was not constructed until 1926. Construction claimed 1,600 lives by 1909 (Benchimol & Silva 2008).

Between 1907-1915, physicians associated with the Rondon Commission combated malaria in the Sertão. The commission was a Brazilian military operation in northeastern Brazil that was to extend telegraph lines from Cuiabá to Santo António de Madeira and then Manaus (Caser & Sá 2011). The semiarid Sertão had river valleys that annually flooded, which allowed some crops to grow, but also provided breeding ground for vectors (Packard & Gadelha 1997, Packard 2007, Caser & Sá 2011). Malaria was endemic and the population was constantly being refreshed with new migrants with no resistance to malaria (Caser & Sá 2011).

While the commission was ultimately successful, disease, chiefly malaria, slowed progress and discouraged settlement by others - indeed, the story of disease and death that emerged from their work dissuaded future migrants in the years to come. Physicians suggested that geography and climate were conducive to reproduction of mosquitoes and the development of diseases including malaria. They blamed the high number of cases on the rubber tappers that lived in poor housing, ate poorly, abused alcohol, overworked and spent long periods in swamps (Caser & Sá 2011).

During the initial years of the Rondon Commission, there were too few physicians to cover the number of staff that became ill. Physicians initially had two support staff each and worked in tents with capacity for 16 men. In 1910, they created a sanitary service for the Rondon Commission. This service would provide the necessary infrastructure to prevent and treat malaria and other diseases. It was be under the control of two physicians that would rotate between the infirmaries of the northern and southern work sites (Santo António de Madeira and Serra do Norte) and the telegraph construction sites every three months. Eight staff members travelled with them. Prophylactic treatment for malaria was provided at the infirmaries. Malaria was prevented through strict control of staff diet, alcohol prohibition and the use of bed nets. At meals, 30-50 cg of quinine salt were distributed at the physician’s discretion. Furthermore, land near the camp would be drained, pools of water filled and mosquito larvae destroyed. Those infected with malaria were to be isolated at the infirmary, which in turn would be brick structures, built on high ground and as far from mosquitoes as possible (Caser & Sá 2011).

Returning to the work of the IOC, Oswaldo Cruz was hired to conduct an antimalaria campaign for those constructing the Madeira-Mamoré Railroad in the state of Rondônia (RO) in 1910. Attempts to build a railroad in the area had started in 1878. The railway was to connect Bolivian rubber production to Porto Velho, RO. It was nicknamed the “Devil Railway” because thousands of workers died of malaria building it between 1907-1912 (Lourenço-de-Oliveira et al. 1989, Deane 1992, Stepan 2003, Benchimol & Silva 2008). The railroad had previously hired American physicians, including five that had served during the Panama Canal construction. Therefore, hiring Oswaldo Cruz was at least partially a political ploy to silence criticism, as he was already respected nationally and international as the director of the Institute and due to his successful campaign against yellow fever in the federal capital. His suggestions echoed the system that the Americans had already put into place (Schweickardt & Lima 2007).

Based at the Candelária Hospital, Cruz examined the conditions of the 113 km of completed railroad and nearby villages along the Madeira River for 28 days during the summer. Oswaldo Cruz praised the railroad for housing staff in Porto Velho because of the city had a functioning water and sewage systems and was built on high ground. Yet health conditions were poor elsewhere. This was partially due to the influx of immunologically naïve Brazilians, but also due to rubber tappers poor diet and the lack of sewage systems and garbage collection in Santo António de Madeira, the base of railroad construction operations. There was little health care outside the camps and the available quinine was expensive and adulterated. Ninety percent of the workers had been infected with malaria and 75% of those had been infected with*P. falciparum* (Deane 1988, Agudelo 1990, Stepan 2003, Benchimol & Silva 2008, Caser & Sá 2011).

Oswaldo Cruz continued to rely upon quinine as a prophylactic, but resistance was reported. He noted that patients could “die of malaria or from quinine intoxication” (Deane 1988, Agudelo 1990, Stepan 2003). His other methods of control were installing house screens and bed nets (Deane 1992). Oswaldo Cruz discarded destroying larval habitat as a control method. He stated that workers continued to fall ill because they did not comply with the measures applied by the sanitation corps. This statement was made despite suggesting that labourers should take daily doses of 2-3 g of quinine a day, which was past the point of toxicity (Benchimol & Silva 2008).

Oswaldo Cruz recommended that railroad labourers not be paid if they failed to take their daily dosage or failed to use their mosquito nets. Those that resisted prophylactic treatment should be fired and those with chronic infection either fired or never hired at all. While the railroad was completed, it is unclear how closely his methods were followed after he left. A report from 1926 suggests they reduced malaria cases from 40-10% and deaths from 15-2%. More recent scholarship suggests the railroad continued to replace workers because of deaths (Benchimol & Silva 2008). After he left, 90% of workers still contracted malaria (Stepan 2003).

In 1912, Oswaldo Cruz returned to the Amazon to create a plan for sanitising the entire Amazon Basin at the behest of the Committee for the Defense of Rubber created by the Ministry of Agriculture, Industry and Commerce. His team, led by Carlos Chagas, travelled along Brazil’s rivers from the coastal city of Belém, in the state of Pará (PA), to the interior of Manaus and visited most major rubber extraction sites. Unfortunately, the team visited the Amazon during the season when most rubber tappers were unreachable in the forest. Yet they still came to a number of conclusions. First, quinine was too expensive, adulterated and rare. Second, Brazilians disliked taking it. Third, new construction in major cities was generating additional vector habitats, which made malaria control difficult (Stepan 2003).

Oswaldo Cruz identified a number of challenges to conducting malaria interventions in the Amazon. One was that its inhabitants were diffusely spread over vast regions and thus communication between them was costly and slow. Another was that rubber tappers worked along the banks of rivers, at times at great distance from one another, as well as far from population centres. Travelling down some rivers was impossible at low tide unless by canoe. Furthermore, rubber tappers barracks were usually located far from the river deep within the forest interior, so they only saw them every 15 days or every month. He proposed that free or low cost quinine should be distributed on a large scale at towns and rubber tapping sites throughout the interior. Chronic infection was common and thus regular treatment provided by small stations was called for rather than hospitalisation (Schweickardt & Lima 2010). The rise of rubber tree extraction in Southeast Asia put an end to the boom in this industry during the early XX century.

The report of Arthur Neiva and Belisário Penna’s trip to the Sertão in 1912 influenced how Brazil’s ruling class viewed people that lived in the Sertão and thus the search for national identity. Their report used images to paint a picture of forsaken people that were resistant to change and marked by backwardness, disease, isolation, illiteracy and poverty. They argued that these issues were not the result of racial inbreeding, an unfortunate debate of the XIX century, but rather due to their suffering from malaria and other avoidable diseases. Sertão’s population was spread thinly over a large area, had no communication with the coast and was lawless. There were few healthcare providers and therefore citizens relied upon folk remedies. Their report was picked up by the journals and newspapers in RJ and magnified by recent claims by Carlos Chagas that disease was the greatest roadblock to Brazil’s progress as a nation. In words of another contemporary figure, Brazil was an “enormous hospital.” It inspired the creation of the Pro-Sanitation League of Brazil, a movement to sanitise the region, advocacy for rural preventative health posts, sanitary education and campaign to federalise Brazilian public health (de Sá 2009).

## Brazil’s first national malaria control efforts

In 1920, the government created the National Department of Public Health (DNSP). Its division of public health focused on Chagas disease, hookworm and malaria (Gadelha 1998). The first health unit of the Rural Prophylactic Service in Baixada Fluminense was run by the mayor of Nova Iguaçu, who later became the Director of the National Department of Health. There was a severe malaria epidemic in the Baixada Fluminense that eventually spread throughout RJ, but the public health response was more successful than previous efforts (Gadelha 1998). Vector and malaria control methods included filling ditches, improved drainage, treating pooled water on riverbanks, improving water ways, oiling water and quinine use (Deane 1992).

For many of the early years of national malaria control efforts, The Rockefeller Foundation (RF) was a crucial partner. From 1922-1925, Mark Boyd and the RF studied malaria epidemiology in the plains surrounding Guanabara Bay, RJ, in the Baixada Fluminense. Their goal was to test if American malaria control methods could be used in the tropics and create guidelines for tropical malaria control to “ascertain a simple, economical and effective method of malaria control adapted for a tropical area, which will offer prospects of permanent relief with a minimum of maintenance”. After a six month survey, Francis Root was the first to describe *Anopheles darlingi*, the likely the malaria vector in the Baixada Fluminense (Deane 1986, 1988, Gadelha 1998). In 1925, the RF and state government reached an agreement to focus work on seven districts where drainage was the most promising malaria control measure (Vincent 1927). Their plan was to spend a year in observation, followed by a two-year campaign of control and maintenance. This consisted of drainage projects combined with the application of Paris green and biological control (Deane 1988, Gadelha 1998).

Another RF staff member, Nelson Davis, studied an outbreak of mountainous malaria in Angra dos Reis, RJ, in 1925. He acknowledged that autochthonous malaria might be propagated by a local bromeliad mosquito, but rejected the hypothesis based on his data (Davis 1926)*.* Elsewhere that year, Arthur Neiva reported to the “Light and Power Company” that the bromeliad vector existed in the Serra de Cubatão and that he had successfully controlled*An. (Kerteszia) cruzi* in Iguape, SP, through deforestation. This contrast in approach underscores the continued disagreement between Brazilian professionals and those from abroad regarding the viability of bromeliad malaria (Gadelha 1994).

In 1926 and 1927, control efforts at the sites covered by Mark Boyd deteriorated because of the foundation sought funding from the municipal government while finances were consolidating at higher levels. In 1927, the RF signed a contract with RJ where 50% of survey costs and 100% of maintenance costs would be covered by the state government (Gadelha 1998). In Itaperuna, no malaria was reported in 1927, two years after work started. In Capivary (sic), drainage was almost complete by 1927 and, compared to a year earlier, half the malaria was reported (Vincent 1927). At the project’s end, no malaria epidemics occurred in the study areas (Deane 1988, Gadelha 1998).

By 1928, the malaria service had been absorbed into the State Department of Health, but the RF continued its collaboration with the state. Efforts shifted to making drainage permanent. In Capivary, Macahé (sic) and Itaperuna, expensive surface ditch maintenance was replaced with subsoil drainage. The seven districts reported a reduction in *Anopheles* breeding and malaria incidence; no malaria was reported in Itaperuna and reduced cases were reported in Queimados, Capivary and Conceição de Macabu. In Carapebús (sic), preliminary survey and field work were conducted in two areas (Vincent 1928). Work continued until 1929. However, the maintenance phase of control broke down due to municipal level disinterest (Agudelo 1990). Despite this, their work would influence future Brazilian malaria control and contributed to local malaria control (Deane 1988, Gadelha 1998)

According to Barros Barreto, who ran the DNSP, “the attempts from 1891 to 1933 to re-establish the former prosperity of Baixada were fruitless” (Gadelha 1998). Furthermore, *An. darlingi *was not just a problem in the Baixada Fluminense. It was established in 1931 that*An. darlingi* was infected with sporozoites in Belém (Mason 1931, Davis & Kumm 1932, Vincent 1933). Infected *An. darlingi* were also reported in França and Itapira, state of Bahia (BA) (Mason 1931).

Greater northeastern Brazil had malaria epidemics during the 1930s. Most land was owned by a few estate owners, often dating back to the XVII century. It was populated with subsistence farmers and sharecroppers, as well as outsiders from nearby towns and seasonal migrant workers from Brazil’s interior. Subsistence farmers from Agreste, which included the states of Paraíba (PB), Pernambuco, Alagoas, Sergipe and BA, often migrated to the coast for seasonal work. Agreste was nestled between the Sertão and the humid coastal plain of the Zona da Mata (Packard & Gadelha 1997, Packard 2007). During droughts, the people of Sertão migrated to the Zona de Mata, Agreste or the Amazon rubber industry. Epidemics in northeastern Brazil were often caused by people from Sertão returning from the interior or coast. For example, a three year drought in Sertão scattered residents until 1935. Returning migrants introduced malaria, which led to an outbreak that began in 1934 and ending in 1937 (Packard & Gadelha 1997, Packard 2007).

## Anopheles gambiae galvanising impact on Brazilian malaria control

The second malaria era started with the introduction of the African malaria vector,*An. gambiae* to Brazil. The campaign it sparked proved to Brazil that an effective national public health response to malaria was possible. Adolpho Lutz visited the port city of Natal, in the state of Rio Grande do Norte (RN) to locate an area free of mosquitoes for a leprosy facility in July, 1928. He made no mention of finding *An. gambiae *and only 28 malaria cases were reported in that year. However, he warned that African mosquitoes could be introduced to the area from Dakar, Senegal. This would be facilitated by mail runs made by seaplanes or fast French destroyers called Avisos that landed near Natal on the Potengí River, starting in March, 1928 (Pinto 1939, Deane 1986, Agudelo 1990, Coura et al. 2006).

Within two years, *An. gambiae* was introduced to Natal from Dakar, most likely by adult mosquitoes that travelled in these planes or ships, as no larvae were found (Mason 1931, Pinto 1939, Packard 2007). By March 1930, Raymond Shannon, a RF entomologist working on the yellow fever, reported there were 2,000 *An. gambiae* larvae 1 km from where the mail vehicles landed (Deane 1992, Killeen et al. 2002). There was a large outbreak of malaria in Natal between April-June (Pinto 1939). By May, all residents were sick with malaria (Deane 1986). The RF predicted that*An. gambiae* would probably remain highly localised, but possibly spread coastally by plane or ship or internally by car and train. It would be particularly a concern in deforested areas (Mason 1931).

In contrast to this conservative analysis, Frederick Soper sent a telegram to the Department of Health that read “Poor Brazil” (Coura et al. 2006). Soper was the director of the International Health Division in South America at the RF (Barber 1940). He had worked on yellow fever elimination since 1927. In 1931, there were 344 deaths in Natal in a neighbourhood near the Avisos docks. In response, the RF yellow fever service helped the Brazilian government eradicate *An. gambiae* in Natal using Paris green. Work ceased in 1932 as Frederick Soper was unable to interest the foundation, state government or federal health authorities in a larger scale *An. gambiae* elimination program [US National Library of Medicine (profiles.nlm.nih.gov/VV/Views/Exhibit/narrative/campaign.html) Agudelo 1990, Packard & Gadelha 1997]. Unfortunately by 1931, prevailing winds spread the vector up the coastline 115 miles, though two dry years kept it from spreading farther (Fosdick 1938).

By 1938, *An. gambiae* had quietly spread from the northwest to the less arid valleys of Assu, Apodí and Jaguaribe Rivers (Deane 1988, Packard & Gadelha 1997, Killeen et al. 2002). This covered localities that were 200 miles to the northwest of Natal (Fosdick 1938). When another drought occurred in Sertão in 1936, it forced migrants into Zona da Mata and Agreste. This led to a predictable increase in malaria cases in 1938 and 1939, but this time the epidemic was magnified by *An. gambiae*. Many of the victims of malaria were from Sertão due to their minimal acquired immunity (Packard & Gadelha 1997, Packard 2007).

During the summer of 1938, a “pandemic” was declared in the state of Ceará (CE) and RN where *An. gambiae* was present. The epidemic had started on the coast and spread up the river valleys into the interior, leading to 150,000 cases and 14,000 deaths in eight months (Deane 1988, 1992, Packard 2007). In some Brazilian states, the invasion of*An. gambiae *was followed in a few weeks by epidemic malaria (Barber 1940). For example, *An. gambiae* was found in October in CE and epidemic malaria was reported in April [US National Library of Medicine (profiles.nlm.nih.gov/VV/Views/Exhibit/narrative/campaign.html)]. Around 40,000 people were infected with more than 20,000 deaths (Agudelo 1990, Killeen et al. 2002). In the Jaguaribe Valley of CE, 10% of the population died. Crops were not planted and salt production was reduced in some areas. It was projected that everyone in the affected areas would be under government relief by 1939 (Fosdick 1938). There were 600,000 cases by the end of the epidemic (Deane 1986).

Towards the end of 1938, officials from the government and the RF met in the epidemic area and decided that an autonomous, well-funded and highly trained organisation needed to be created to eradicate *An. gambiae *(Gusmão 1982). They also concluded that, once*An. gambiae* reached a river valley, it would spread throughout unless blocked by a natural or artificial barrier (Fosdick 1938). Along the coast, *An. gambiae* spread at an average speed of 40 miles a year, most likely by boat. Yet without rivers to follow, the arid interior blocked its progress. In retrospect, Soper claimed that 15 or 20 men could have blocked its entrance into the narrow alluvial passages that gave it access to the interior (Fosdick 1939).

According to the journal Gazeta de Notícias, CE, “human language is…[in]adequate to describe the desolation…in which suffering, tears and mourning spread their lugubrious mantle over thousands of graves. The general belief was that the Northeast would be depopulated because those that did not die at once would abandon it” (Killeen et al. 2002). These events put malaria on the national stage and raised the spectre that *An. gambiae* might reach the Panama Canal (Deane 1992, Packard & Gadelha 1997, Packard 2007).

In August 1938, President Getúlio Vargas created a new emergency antimalaria service (Killeen et al. 2002). In January 1939, the president decreed the creation of the Northeast Malaria Service (Agudelo 1990). By then, *An. gambiae* had spread 300 miles to the west of Natal and was found in 12,000 mi^2^. Federal governmental funding went from $250,000 in 1938 to $500,000 in 1939 with the RF providing $100,000 in 1939 and $230,000 in 1940 (Fosdick 1939).

While the Northeast Malaria Service focused on malaria, Frederick Soper pushed the RF to focus on confining *An. gambiae* to arid regions, or even eradicating it, because he believed it was a threat to North and South America (Fosdick 1938, Packard & Gadelha 1997). The RF assisted in the financing and organisation of the service using the infrastructure created for the yellow fever service ($3,200,000 dollars in 1995 dollars) (Killeen et al. 2002). It was willing to pay 20% of the service’s costs, if it had direct control (Fosdick 1939). Frederick Soper ran this service from 1939-1941 along with Paulo Antunes, 70 physicians and 4,000 guards (Agudelo 1990,Deane 1992, Killeen et al. 2002).

Initial efforts were “disappointing” because the service started at the beginning of the rainy season when *An. gambiae *spread most rapidly. The rain led to widespread malaria epidemics and the treatment of 114,000 people. Nonetheless, there was a measurable reduction in mortality. By July, the service had a staff of 2,000 physicians, technicians, scouts, inspectors, guards and labourers. Despite a prolonged rainy season, the spread of the vector had been minimised and vectors disappeared from some previously heavily infested areas. The extended dry season was a boon to the *An. gambiae *control program (Fosdick 1939).

Frederick Soper framed his efforts as a war, with fumigation posts on outgoing roads, likened to the Maginot Line. His “scorched earth” was a 10-mile perimeter around*An. gambiae* range limits, which was to be kept noninfectible. The entire area was mapped from the air to ensure no collections of water were left untreated with larvicides within the perimeter or the infected zone (Fosdick 1939). Staff also used atebrine and sometimes quinine (Silveira & de Rezende 2001). Homes were sprayed with insecticides and every car and train that left the area was fumigated, as well as every boat and plane disinfected before leaving for uninfested ports. This did not always work; at least one unfumigated car took a backroad and spread *An. gambiae* miles into uninfested territory. Another time, a fishing boat spread it up the coast. Still, by December,*An. gambiae* was pushed back to the main river valleys and the coast. There was optimism that it might be eradicated (Fosdick 1939).

Cases began to diminish and they treated 30,000 in January 1940 and just 400 in September (Gusmão 1982). After the rainy season, *An. gambiae *was restricted to the lower Jaguaribe Valley, though another small population was found in October, 60 km from the infested area (Fosdick 1940). The last *An. gambiae* larvae was reported in November 14 1940 (Agudelo 1990, Killeen et al. 2002).

Control efforts were aided by *An. gambiae *indoor-biting behaviour and the Northeast dry season, during which it was difficult for mosquitoes to locate breeding grounds (Barber 1940, Killeen et al. 2002). They were also aided by Frederick Soper’s use of staff that had previously worked on the *Anopheles aegypti* campaign and an almost militaristic approach to program administration (Deane 1988). Finally, recent molecular work suggests that the invading vector was actually *Anopheles arabiensis*. This species is adapted to arid climates and therefore it may not have been able to invade the wetter regions surrounding northeastern Brazil (Parmakelis et al. 2008).

In a memo to the RF in 1942, Fredrick Soper said: “The eradication of *An. gambiae* from…Northeast Brazil is an important event in public health….because it relieves the continent of the immediate threat…[and] dramatically calls attention to the possibility of controlling mosquito-borne disease through species eradication.”. Frederick Soper later said that the success in eradicating*An. gambiae* rehabilitated the concept of malaria eradication. His success, Arnoldo Gabaldon’s in Venezuela (Griffing et al. 2014) and others led to a shift from the holistic approach put forth by the League of Nations Malaria Commission to Malaria Eradication through vector control (Packard 2007).

Fredrerick Soper’s influence ended when he was called away to World War II (Deane 1986, Agudelo 1990). However, the end of his campaign did not mark the end of*An. gambiae* attempts to invade South America. By 1941, commercial aircraft leaving Africa were sprayed before leaving and again upon arrival in Brazil. Dead *An. gambiae* were reported on planes in October 1941 and January 1942 (Fosdick 1941). In 1943, live *An. gambiae* were reported on planes arriving from Africa and mosquitoes were found in homes near Natal airport. The Brazilian government invited a RF staff member to review protective measures and resurvey the formerly infested area. The report recommended that emergency landing fields in Brazil needed to focus more on “disinsecting” transatlantic planes on arrival (Fosdick 1943). After these efforts, the RF generally fell silent on malaria. They did, however, fund an entomological laboratory at the Malaria Institute in RJ in 1951, where insecticides, herbicides and molluscicides were tested. They also investigated how to spray bromeliads (Barnard 1951).

## The shift to national Brazilian malaria control

During the 1940s, there were four-six million malaria cases a year among 45 million Brazilians. More than 50% of cases occurred outside of the Amazon (Deane 1986, 1988, Coura et al. 2006, Packard 2007). Some cases were due to the government enlisting 50,000 northeasterners as “rubber soldiers” (Deane 1986, Lourenço-de-Oliveira et al. 1989, Coura et al. 2006). Outside the Amazon, valleys with large rivers supported malaria including São Francisco, PR and the Baixada Fluminense (Deane 1986).

There were three organisations involved in Brazilian malaria control: the São Paulo State Antimalarial Service, the Special Public Health Service in the Amazon (SESP) and the National Malaria Service (SNM) (Deane 1988, Silveira & de Rezende 2001). SESP was founded in 1942 with financial support from the Brazilian government and the US Institute of Inter-American Affairs (IAIA), which was then coordinated by Nelson Rockefeller and advised by RF staff. SESP was partially the product of a meeting of the Ministries of Foreign Affairs of the American Republics that was held in RJ after the invasion of Pearl Harbor. The United States of America needed raw materials for the war effort, particularly rubber from the Amazon, after the Japanese cut-off supplies and iron from the Rio Doce Valley, after the Germans cut-off European supplies. The goals of SESP were (i) to provide sanitation for the Amazon Basin, mostly through malaria control and health care for rubber tappers and (ii) to provide training for physicians, nurses and sanitary engineers. Eighty percent of SESP’s funding came from the IAIA until 1944, when Brazil began to nationalise it. While SESP was nominally under the Ministry of Education and Health, in practice it was autonomous. Belém was the centre of field operations and training (de Campos 1998, 20, 2008).

Though the Americans viewed SESP as a temporary wartime program, the Brazilian government viewed it as a convenient tool for the expansion of economic activity and public authority that did not conflict with the existing public health agenda. Effectively, the IAIA subsidised the creation of a network of health facilities in the Amazon focused on malaria control and health care for rubber tappers, as well as the expansion of state power into the Amazon and the Rio Doce Valley. In the Northeast, a few cities received additional malaria control around American bases (particularly, Belém, Recife and Natal). They identified the 30 most important populations centres in the Amazon, established headquarters for health districts, then divided these health districts into subdistricts and then zones managed by sanitary inspectors once a week. SESP gave 7.7 million tablets of atebrine to rubber tappers across the Amazon Basin by 1946. However, their methods were insufficient to control malaria in the Amazon. SESP also provided malaria control in that part of the Rio Doce Valley had iron, mica and quartz mining sites, as well as a railroad in disrepair - but only after the RF refused to run the program. This program was successful, partially because the area was so much smaller than the Amazon (de Campos 1998, 1999, 2008).

The SNM was created in 1941 and was run by Mario Pinotti starting in 1942 (Deane 1988). In 1943, the SNM began to study the life history, distribution and density of the *An.*(*Kerteszia*) complex in southern Brazil. “Bromeliad malaria” covered 40,000 km^2^ and thus one million Brazilians across the coasts of the state of Paraná (PR), northern the state of Rio Grande do Sul, SP and SC. Furthermore the vector was present from PB to RN, but only the South had a sufficient density of bromeliads to support malaria endemically. They found high densities of bromeliads in major cities like Florianópolis, small towns like Brusque and Joinville, as well as harbours, resorts and beaches near rainforests. This meant bromeliad malaria, or “forest malaria” was effectively an urban concern. It was also highly domestic; 99.2% of the 20,000 mosquitoes captured in homes in 1944 and 1945 were *Kerteszia* species, which bit during all hours, inside or outside the home (Gadelha 1994).

In response, 20 million bromeliads were destroyed amongst the houses nestled in the low rocky areas of Florianópolis between 1944-1947. This eradicated malaria from the city. However, it was dangerous and tedious work and mosquitoes still flew from faraway forests into cities. Therefore control efforts shifted to deforestation and less commonly to herbicides, particularly copper sulfate. A recommended deforestation method was to remove trees 500-1,000 m from towns and then plant two-thirds of that land with vegetables and flowering plants and one third eucalyptus. This trend continued through 1950 when autochthones cases were considered rare. Herbicides, chemotherapy and DDT would eventually replace deforestation as the preferred control method. However, the vector’s tendency to bite wherever and whenever it liked limited the benefits of DDT domicile spraying, which led to the resurgence of deforestation as the preferred control method by the 1970s (Gadelha 1994). Later scholarship reached the consensus that *An. cruzi*, *bellator* and*homunculus* were the vectors responsible for bromeliad malaria (Deane 1986). Internationally,*An. (Kerteszia) cruzi *was finally acknowledged as a potential vector due to work by the US Army in Trinidad (Rozeboom & Laird 1942).

## Brazilian malaria after the introduction of DDT and chloroquine (CQ)

DDT was first used in Brazil in an organised fashion in Breves, PA, by the SESP in 1945. Houses were sprayed every two months and later every four. CQ tablets were given out to doctors, malaria inspectors and influential locals to distribute to malaria patients. Malaria incidence drastically decreased. Later, CQ was distributed at hospitals in Belém and Santarém with total doses of 1,500 mg (Reyes 1981, Deane 1988).

Due to the success of this effort, DDT was systematically used in the state of Amapá (AP), AM (including Manaus), RO (Porto Velho in 1946, other villages in 1947 and the railroad in 1948) and PA (including the Bolonha and Água Preta dams) to reduce*An. darlingi* populations (Deane et al. 1948, Lourenço-de-Oliveira et al. 1989). In Belém and Manaus, spraying included houses, cinemas, churches, theatres and night schools (Deane et al. 1948). By 1950, the SNM had moved into the Amazon and managed control everywhere except in SP. Malaria had been wiped out in the Northeast, decreased on the coastal plain and was reduced in the Amazon. By 1954, DDT spraying covered regions occupied by three million Brazilians (Deane 1988).

In 1956, Mario Pinotti became the director of the National Department for Rural Endemic Diseases, a newly created department in the Ministry of Health that centralised public health activities against many endemic diseases including malaria, yellow fever and Chagas disease. He changed the SNM into the Malaria Eradication Campaign (CEM) in 1957 (Deane 1988) in response to the World Health Organization (WHO) establishment of the Global Malaria Eradication Program in 1955. CEM decided to put CQ in table salt in areas where insecticides were difficult to apply. This was called “Pinotti’s method”. After early 1950s trials, chloroquinised salt was widely distributed in Brazil (Payne 1988). Initially, a fine CQ powder was added to coarse salt in 30 kg bags. After adjusting the mixture to avoid CQ settling, chloroquinised salt was successfully used by the Industry and Trade of Ores S.A., mining company in AP (Gusmão 1982,Deane 1988).

As Mario Pinotti’s political career began to fade in 1960, so too did the use of chloroquinised salt due to concern that targeted populations were unevenly protected (Hochman 2008). In AM, chloroquinised salt distribution was no longer required, but it was distributed until supplies ran out (Póvoa 1993). Still, chloroquinised salt continued to be used in French Guiana (1967-1971), Guyana (1961-1965) and Suriname (1966-1972). It has been argued that chloroquinised salt may have encouraged early parasite resistance (Payne 1988).

CEM reached the state of SP in 1959. Malaria was common throughout the state from the 1930s-1950s. People migrated from the countryside to urban centres and the state shifted from agriculture to industrialisation (Alves et al. 2004). During the 1930s-1950s, SP malaria control initially consisted of small, ineffectual engineering projects to control vectors, with funding often coming directly from land owners. It then shifted to focal monitoring. Breeding sites and larvae involved in transmission were identified and larvicides were applied. The impact of these efforts on *An. darlingi*populations were limited because they were time consuming and the number of potential breeding sites overwhelming. Control efforts were limited to infrastructure, agribusiness and economically important urban environments (Barata 1998).

CEM began in 1959 and focused on the 198 municipalities that had malaria. The attack phase of control occurred between 1960-1963 and consisted of spraying houses with DDT every six months. Inhabitants with fever were actively sought out and 10% of the population was tested for malaria each year. In addition, 5,000 posts were created for passive surveillance and malaria treatment. By 1968, 68% of the population was living in a malaria-free zone, with much of the remainder in the consolidation phase (31%). It took four years to completely interrupt malaria transmission (Barata 1998).

In AP, a mining company established a community named Serra do Navio around 1954. By 1960, CEM was providing AP with chloroquinised salt, but starting in 1963 the company began to provide miners and all other inhabitants with chloroquinised salt in Serra do Navio, Porto Platon and Vila Amazonas. They continued to do so for the next 30 years. Chloroquinised salt led to a sharp decrease in malaria infections with no cases reported in Serra do Navio by the early 1990s. In the 1960s,*An. darlingi *was found in residential areas, but the company’s malaria control program, which included biannual DDT spraying, locally eradicated*An. darlingi* for the next 40 years (Póvoa 1993, Póvoa et al. 2001).

DDT spraying was also officially undertaken throughout the Amazon in 1960 (Hochman 2008). During the 1960s, malaria cases were confined to the Amazon. Remaining transmission was attributed to (i) the dispersed Amazon population hindering control, (ii) housing that lacked or only had partial walls, which facilitated vector contact and hindered DDT spraying and (iii) CQ resistant *P. falciparum* (Barata 1995).

Different Amazon habitats had different malaria profiles. The rubber plantations had low population density and mobility. DDT application and bed nets were beneficial and reduced transmission. In the pastures, there was also low incidence due to low worker density and little *An. darlingi* habitat. Construction camps were generally free of malaria due to vector control, early diagnosis and treatment. “Closed” prospectors were free of malaria because their working environment was unfavourable to transmission, but opencast prospectors actually created ideal vector habitat and thus worked in high malaria incidence areas. Furthermore, they worked in few clothes during peak vector activity periods and there were many asymptomatic carriers. The leading edge of Amazon colonisation was also a high-risk zone due to the presence of ideal *An. darlingi* habitat where deforestation was occurring. Amerindians had more or less malaria incidence depending on their exposure to “the white man” (Marques et al. 1986, Packard 2007).

Until 1960, the USA had continued to renew the agreement SESP was based upon due to the Cold War. SESP activities shifted to highlighting the positive impact of cooperation between the two nations by creating “human capital” for Brazil’s development plans, instead of just areas that produced materials Americans needed. SESP became the provider of public health services to regions Brazil wanted to economically develop, including the São Francisco Valley and the Amazon. SESP lost its autonomy when it lost American funding and became the Public Health Special Service Foundation (FSESP) under the Ministry of Health (de Campos 2008).

**Fig. 1:**
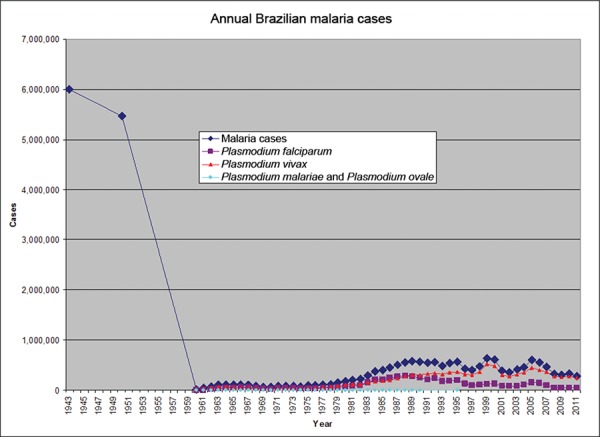
: annual estimated malaria cases in total and by species, when available. Estimate quality likely varies based on multiple unmeasured factors. Numbers reported for 1991-1994, 1996-1999 and 2009-2011 were based on visual estimates from line and bar graphs and are reported for qualitative analysis. The two early data points may be suspect (Coura et al. 2006, PAHO 1991, 1996, 2010, SIVEP-Malaria 2014).

**Fig. 2:**
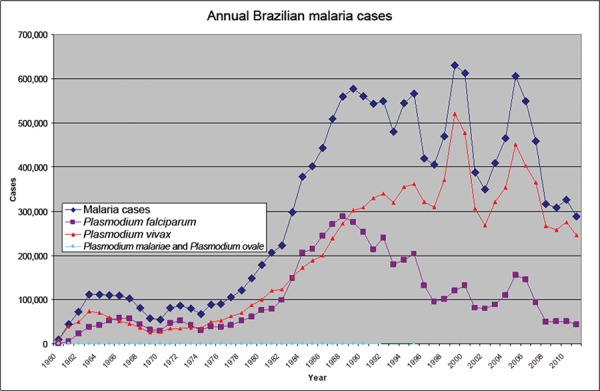
: this figure represents the same data as Fig. 1, with the same disclaimers. However, the two earlier estimates have been removed.

During the 1960s, the government began to secure Brazil’s borders by colonisation and the construction of roads linking the North with other regions (Marques et al. 1986, Packard 2007). Manaus and Porto Velho had epidemics due to the influx of immigrants from the Brazilian Amazon and the creation of slums where mosquitoes could breed (Barata 1995).

## Brazil adopts the CEM

In September 1965, the Brazilian government adopted a CEM model recommended by the WHO. CEM was financially and administratively autonomous and relied upon DDT spraying and antimalarial distribution where malaria was present. CEM eliminated malaria from the Northeast, the Southeast, the Central-West and the South (Moran 1993).

As the number of malaria tests increased to 1.7 million per year by 1969, the number of positive tests diminished. However, control efforts did not eradicate transmission outside of the home and *P. falciparum* began to show signs of CQ resistance. Additional factors that led to the failure of CEM included the absence of social and health infrastructure and the presence of at risk populations of miners, agricultural colonists and loggers that were often exposed to vectors (Loiola et al. 2002, Coura et al. 2006).

CEM was suspended in 1970 due to decreased cases, criticism of the public health administrative model and concerns over DDT brought on by the book Silent Spring (Hochman 2008). There were only 28,557 cases of *P. falciparum* in a population of 92.3 million Brazilians in 1970 (PAHO 1991, Barata 1995). Of these, 73% of the remaining cases occurred in the Amazon due to its climate and wall-less dwellings (Deane 1988). Malaria had been reduced to 1% of its incidence in 1950 (Stepan 2003). There were less than 100 autochthonous malaria cases each year in SP (Barata 1998).

## Malaria control is integrated into public health services and malaria increases

During the 1970s, the Superintendent of the Public Campaigns of Brazil (SUCAM) (which evolved from the National Department for Rural Endemic Diseases) integrated the malaria eradication programs with other programs, including the general census in 1970, the smallpox eradication program in 1971, the action against meningococcal meningitis and Chagas disease in 1975 and the campaign against leishmaniasis and leprosy notification in 1976 (Agudelo 1990). This integration of malaria eradication with other public health programs reduced the resources available for malaria control (Loiola et al. 2002).

In 1974, all Brazilian malaria regions had been through the attack phase and there were only 50,000 cases (Barata 1998). Of these, 24,000 cases were registered in states of Acre (AC), Maranhão (MA), AM, PA and more than 12,000 occurred along the Trans-Amazonian Highway and its surroundings (Gadelha 1998). By 1976, malaria was under control in the South, Southeast, Northeast and part of the Central-West. Malaria was only prevalent in the Amazon and Northeast (Coura et al. 2006). Yet malaria transmission began to increase, including in all of the areas where malaria transmission had been interrupted, due to more Brazilians moving to the Amazon for colonisation and the construction of hydroelectric projects and roads (Marques et al. 1986, Barata 1998). DDT resistance was reported by 1975 in Brazil and Colombia (Agudelo 1990).

In southern PR, autochthonous cases were reported in outbreaks that occurred in 1976 (150 cases), 1977 (296 cases) and 1978 (413 cases), predominantly on the coast. Malaria also occurred to the Northeast, just north of Guaira, among the islands of the Paraná River and in the municipality of Querência do Norte. There were fewer cases in 1979 (Bértoli & Moitinho 2001).

## The impact of migration and colonisation on malaria incidence

Prior to the 1970s, there had been little Brazilian Amazon deforestation (Moran 1993). As late as 1975, only 0.6% of the Amazon had been cleared (3,000,0000 ha). The rate of Amazon deforestation increased between 1975-1987 (with 8,000,000 ha cleared in just that year) (Moran 1993).

During the 1970s, the National Institute for Colonisation and Agrarian Reform convinced Brazilians to move from the South and Southeast to Amazon Region. There were two colonisation programs: the Organised Settlement Programs, which focused on skilled farmers, and the Integrated Colonisation Projects (PIC), which focused on the landless poor (Silva-Nunes et al. 2006). Roads were constructed in the Amazon and the development of small and medium-sized farms was encouraged continuing into the 1980s. There was an immense migration of Brazilians from the Northeast, Central-West and South into the Amazon, including RO, that were unaware of how to protect against malaria and immunologically naïve (Loiola et al. 2002).

In RO, there were 116,000 inhabitants and the annual parasitological index (API) was 50/1,000 in 1970 (Salcedo et al. 2000). The Brazilian government began a development plan that encouraged southerners to create farms in RO by giving them cleared land. As the land became exhausted, settlers shifted to cattle ranching or sold their land (Takken et al. 2005). Over the decade, 277,000 immigrants arrived (Camargo et al. 1999). By 1980, there were 492,810 inhabitants in RO and 96% of malaria cases occurred in the Amazon Region (Deane 1988, Lourenço-de-Oliveira et al. 1989). The city of Ariquemes was the centre of Rondonian deforestation (Takken et al. 2005).

Previously, Brazilians had moved from the North/Northeast to the South, but between 1970-1980 movement reversed (Gadelha 1998). In total, one million Brazilians moved into the Amazon, with the majority going to RO and other gold mining areas (Takken et al. 2005, Packard 2007). The construction of roads, hydroelectric plants, mines and livestock and agricultural projects led to increased malaria transmission. Malaria cases increased from 52,469 in 1970 to 169,871 in 1980 (Marques et al. 1986, Barata 1995). In 1980, 97.5% of malaria was still confined to the Amazon Basin [34.8% occurred in RO, 22.4% in PA, 11.3% in MA, 9% in MT and 8% in the state of Roraima (RR)] (Barata 1995). Outside of the Amazon Basin, most cases occurred in the state of Goiás (GO), followed by PR, SP and the state of Mato Grosso do Sul (MS) (Barata 1995).

Unfortunately, malaria carriers spread throughout Brazil in a dizzyingly array of directions in the 1980s. Cases came from the Amazon states and constantly presented the risk of the reintroduction of autochthonous malaria (Barata 1995). AC had many imported cases arriving from RO, with a few coming from AM. AM received cases from gold miners arriving from RO and RR*.* AM miners also spread malaria to Itaituba, PA (Gadelha 1998). Other miners entered PA from northern MT, particularly from the mines around Peixoto de Azevedo River. MA received numerous cases from PA and MT. Cases from RO spread to MT as well. GO received cases from PA, MT and MA. RR received cases from multiple Amazon Basin states including RO, AM and MA (Gadelha 1998).

During the 1980s, the majority of malaria cases occurred in Amazon municipalities with mining and agricultural projects (Barata 1995). Important vectors included *An. darlingi* and*Anopheles albitarsis* (Póvoa 1993). The National Institute of Colonisation projects called for deforestation of three or four acres in the first year followed by a controlled burn and then planting rice, corn and beans. Colonists lived in temporary camps in plastic tents or palm huts near the water, which favoured malaria transmission. At the Machadinho Project, 90% of colonists had malaria at least once after their arrival and the index of infection among* An. darlingi* was 5.2%. At many mines, malaria was resistant to control due to makeshift housing and the constant movement of miners (Barata 1995). Hundreds of thousands of gold miners entered and exited the interior and thereby spread malaria throughout Brazil (Marques et al. 1986). There were multiple migratory channels in Brazil including: southern PA and northern MT to MA, northern MT to SP, PR, GO and MS and RO to SP, PA, AC and AM (Barata 1995). The largest migration was into RO from everywhere, but the North, followed by northeasterners moving into PA. Most settled along the Trans-Amazonian Highway or at mining locations (Marques 1987).

## SUCAM focuses on Amazonian malaria control

SUCAM focused its attention on the Amazon during the 1980s, particularly in areas with hydroelectric, mining and subsistence farming projects. It applied DDT indoors, eliminated mosquito breeding grounds and supplied antimalarial drugs, as well as technical supervision. It abandoned CEM in favour of nuanced local projects with epidemiological stratification and “microzoning” (Barata 1995). It divided the Amazon Region into priority areas I and II based on risk factors that could impact epidemiology (Coura et al. 2006, Atanaka-Santos et al. 2007). Priority I regions had “frank” transmission and were examined by SUCAM district boards for case classification and provenance identification. Priority II regions had low intensity, stable transmission and cases were to be investigated for identification and classification of local transmission when possible (Marques et al. 1986). The new approach was first used in PA and RO (Loiola et al. 2002).

New techniques were used, including outdoor ultra-low-volume nebulisation, mass treatment, impregnated curtains and new insecticides. In other areas, including colonisation areas in RO, control efforts remained unchanged despite their failure (Barata 1995). Traditional control methods were ineffective in states like RO, in communities along the Amazon River. In such communities, screening homes was ineffective because walls were porous and allowed air flow, sylvatic vectors were behaviourally resistant to DDT domicile spraying and inhabitants would have to stay under bed nets between 05:00 pm-06:00 am. This left patient care as the only viable control method, which might prove ineffective due to asymptomatic cases (Alves et al. 2005).

## Malaria cases increase in the Amazon Region

From 1980-1985, malaria cases increased 2.4 times (Barata 1995). In AC, there were 5,000 participants in the settlement program in 1981 and 33,600 by 1985, while the population of RO went from 570,000-1,040,000. PA’s mining areas doubled in population, due to the influx of immigrants from the state of Piauí (PI), MA, GO and CE (Marques 1987). In 1983, 98% of malaria cases in SP were introduced and cases occurred in farm workers, miners and truck drivers (Barata 1998). By 1984, 92% of the 205,000 cases of malaria were reported among the 12 million people living in the northern and western Amazon and the remainder were exported from these regions (PAHO 1984). New foci of infection were found in PI, CE, BA, MG, MS and RJ (Packard 2007). In 1985, 86.4% of Brazilian malaria cases were in PA and RO and restricted to mining, riverine and settlement areas (Oliveira Filho 1992).

In 1986, 99% of malaria cases occurred in the Amazon (Deane 1988). Autochthonous cases predominated in the Amazon states, with movement between them leading to imported cases as well. RO, RR and AP had the highest autochthony indexes and were also the least influenced by neighbouring states. Many of the cases reported in AC came from RO and the border with Bolivia. In AM, most cases from Manaus and the middle of the state were imported, but autochthonous cases predominated elsewhere. In Belém it appeared that the neighbouring AP and MA contributed cases. In GO, most cases came from the municipalities of PA and MT. Cases in MT were generally autochthonous though some cases came from RO (Marques et al. 1986). Malaria cases began to increase in the southern state of PR (Bértoli & Moitinho 2001).

In the Northeast, Southeast, South and a portion of the Central-West, most cases were introduced from the Amazon. The one exception was BA, where autochthonous cases and introduced malaria (mostly from RO) were present. While there were autochthonous cases in the northeastern states of PI and CE, returning migratory workers also introduced malaria. In the Southeast, there were few autochthonous cases with the majority imported from RO and secondarily PA. In the South, cases were almost all imported, particularly from MT and RO. Autochthonous cases were registered in two municipalities along the SC coast and in western PR on some Paraná River islands. In general, malaria outbreaks did not occur in locations with major infrastructure or where migrants were under the direct control of big business or the government (Marques et al. 1986).

During a few months in 1986, the government mobilised human and financial resources to rapidly reduce the morbidity and mortality associated with malaria in MT, PA and RO in “Operation Impact.” At the time, these states contained 80% of Brazil’s malaria. Approximately 2,000 staff were transferred to cover these regions. Mefloquine treatment of *P. falciparum* was introduced to treat resistant *P. falciparum* cases, but CQ, primaquine and sulphadoxine pyrimethamine were also used. Operation Impact’s outcome did not justify the high resource cost, nor the indiscriminate use of mefloquine in hyperendemic areas (Loiola et al. 2002). Malaria cases went from 52,469 cases in 1970 to 559,535 in 1988 (Brazilian Ministry of Health, unpublished observations).

By 1988, Amazon deforestation had declined because of a recession and hyperinflation (Moran 1993). During the 1940s,*An. darlingi* had made up 26% of the anopheline species composition, but it was 77.7% in the 1980s and greater than 90% in the 1990s.*An. darlingi* behaviour seems to have become less endophilic as well (Gil et al. 2003).

## The government responds with the Amazon Basin Malaria Control Project (PCMAM)

In 1989, the Brazilian government asked the World Bank to fund the PCMAM due to the increasing problem of malaria and regional political pressure. The World Bank provided Brazil with $99 million dollars and the government provided another $99 million. The funds were to be used over the next five years. The goals of PCMAM were (i) to reduce malaria’s occurrence, (ii) to develop SUCAM and the state secretariats of health and (iii) to focus attention on the health of indigenous communities. PCMAM also developed local public health services with regards to diagnostics and treatment (Loiola et al. 2002).

In 1980, all homes in malaria-affected areas were sprayed with residual insecticide, but PCMAM shifted to limited insecticide application to the minimal number of houses required. PCMAM focused on applying insecticides through outdoor ultra-low volume application and thermonebulisation, particularly in AM. While selective vector control was an important policy advancement, in practical terms there was not enough staff for implementation (Loiola et al. 2002).

In 1990, Amazon had 99% of Brazil’s malaria cases, 85% of which occurred in just 79 Amazon municipalities (PAHO 1991, Barata 1998). Disease spillover from the Amazon most affected SP and PR because of most individuals leaving the Amazon were diagnosed and treated in those states. In SP, 15 municipalities had transmission, while 224 had imported cases (Barata 1998). Agricultural practices in AC and RO also encouraged malaria cases (PAHO 1991). Malaria was most prevalent in RO (33.4% of all cases among 1,130,000 inhabitants and an API of 216.7/1,000), PA (20.1%) and MT (25%), where miners were active (PAHO 1991, Salcedo et al. 2000). The principle Amazonian vector was *An. darlingi, *but on the coast it was *Anopheles aquasalis* (PAHO 1991, Dourado 1992). The government did not provide RR with public health care until 1991 (Chaves & Rodrigues 2000).

Mining and public works continued to encourage malaria as exemplified by Peixoto de Azevedo, MT, and the Itaipu reservoir, in PR. Peixoto de Azevedo was founded in May 1986 after the discovery of gold and became home to ~30,000 miners. As the price of gold dropped and it became more difficult to mine, the miners relocated, with some moving to Leonislândia to start small farms in 1993. In 1996, Leonislândia had 4,000 inhabitants, with homes built close to the forest and near streams. These conditions led to the quick establishment of malaria and *An. darlingi* (Duarte et al. 2004).

The Itaipu reservoir led to malaria cases between 1994-1999. Cases were attributed to movement of hosts between the Brazilian Amazon and Paraguay and new mosquito breeding grounds at the Itaipu Lake. The National Health Foundation (FUNASA) had undertaken household spraying and reduced cases starting in 1990, but the reservoir created a new epidemic in 1995. Other issues that contributed to the increase in malaria cases included the proximity of towns to other reservoirs that could support*An. darlingi*, the development of professional fishing and leisure activities around the reservoirs and homes that were typically wooden huts that allowed vectors inside and made insecticide spraying difficult (Bértoli & Moitinho 2001).

## PCMAM paralysed

In 1991, the Ministry of Health combined SUCAM with FSESP, the National Secretariat of Special Health Programs and National Secretariat of Basic Health Actions into the FUNASA*.* As a result, PCMAM was “practically paralysed” between 1991-1993. During this time, private control efforts were also conducted. In the municipality of Calçoene, AP, a mining company started malaria control efforts in 1991 with help from the Evandro Chagas Institute and FUNASA*.* At the beginning of the 1980s, Lourenço, the district where Calçoene was located, had greater than 50% of the malaria found in AP. The mining company’s program involved improving the quality of hospital facilities, laboratories, vector vigilance and control and training of personal (Couto et al. 2001).

Given the difficulties implementing PCMAM, it was extended for another three years, though the funding for the program was reduced. The Integrated Malaria Control Program was created, which was effectively PCMAM adjusted to account for recent WHO strategies, particularly early patient diagnosis and timely and appropriate treatment (Coura et al. 2006). With PCMAM back on track, the Brazilians increased the diagnostic laboratory and treatment network and the number of doctors and support staff (Loiola et al. 2002). They also made malaria treatment widely available through mining area shops and all fevers were presumptively treated. CQ was the first line treatment and mefloquine was the second (Barat 2006). From 1989-1996, 81% of malaria cases were *P. vivax* and 17.1% were *P. falciparum* (Salcedo et al. 2000, Martínez-Espinosa et al. 2004). The incidence of malaria began to diminish in 1989 due to control strategy changes and a shift in colonisation zones to occupation rather than deforestation (Barata 1995). PCMAM reduced malaria from 577,787 cases in 1989 to 221,600 cases in 1996 and the mortality rate went from 7/1,000 in 1988 to 1.8/1,000 in 1995 (Loiola et al. 2002, Packard 2007).

## PCMAM falters

Unfortunately, as PCMAM funding depleted, malaria resurged. FUNASA created a new plan to intensify malaria control at the end of 1996, which focused on 100 municipalities with APIs of greater than 50/1,000 and in some municipalities and state capitals where malaria was a serious problem despite lower APIs. Decentralisation of the public health response was also attempted with mixed results; while municipalities were involved in the process, the role of state government was not adequately valued and therefore there was no local funding when federal money ran out (Barat 2006).

Between 1998-1999 there was a 26% or 34% increase in malaria cases, which demonstrated the instability and fragility of the surveillance system (Barata 1995, Loiola et al. 2002). In 1999, there were 631,000 malaria cases, of which 99.7% occurred in the Brazilian Amazon (Duarte et al. 2004, Packard 2007). During the 1990s, malaria was in all the Amazon states. In AM, the highest API occurred around Manaus among migrants in poor sanitation areas where vectors could breed. There were 21,234 malaria cases in Manaus in 1997 and 83% were *P. vivax* (Martínez-Espinosa et al. 2004). In PA, the highest APIs were associated with mining and colonisation projects (Póvoa et al. 2001). In AP, cases occurred in important mining areas and *Anopheles marajoara*was reported as a new emerging vector (Barata 1995, Póvoa et al. 2001). In RR, the high APIs occurred where mining was taking place (Barata 1995). In western MT, malaria was hypoendemic, as in most of the Brazilian Amazon and the highest number of cases occurred in areas with mining areas (Barata 1995, Duarte et al. 2004). In AC, cases were localised near agricultural and rubber plantations around Abunã River (Barata 1995). In RO, the high APIs occurred where mining and colonisation projects were taking place. By 2000, deforestation had reached Porto Velho. Cattle replaced farm crops. These factors encouraged*An. darlingi* population increases (Takken et al. 2005).

## The government introduces the Program for the Intensification of Malaria Control (PIACM)

The Brazilian Government announced in October 1999 that they would reduce malaria cases by 2001 to half the number seen in 1999 and half the mortality by 2002 at the first international meeting of the Roll Back Malaria program of the Pan American Health Organization (PAHO) in Peru. They would do this through the PIACM in the nine-state legal Amazon. PIACM started in 2000 and cost $50.2 million (Loiola et al. 2002, Takken et al. 2005, Silva-Nunes et al. 2006).

PIACM had new elements: (i) political involvement at all levels of government, (ii) regional development, (iii) an assessment of the social development cost of malaria, (iv) integration of related government offices including the Ministries of Health and Agrarian Reform, (v) a structured service strategy, (vi) periodic assessments of progress and (vii) a guarantee of consistent funding from all levels of government (Loiola et al. 2002). Vector control was expanded with more equipment, vehicles and personnel. This allowed for increased indoor insecticide spraying and the spatial treatment of outbreaks. Drainage projects in urban centres including Manaus and Porto Velho were also undertaken. In addition, it was decided that all new settlements would have to be extensively evaluated for malaria prevention (Takken et al. 2005).

In 1999 there were 630,985 malaria cases, but by 2001 there had been a drastic reduction to 383,654 cases. *P. falciparum* cases were reduced by 35% and *P. vivax* cases by 41%. Such a reduction had not occurred in the last 41 years and was attributed to the implementation of PIACM in 2000 in most states, its implementation in 2001 in AP and a similar AM plan that had been implemented in 1999 (Loiola et al. 2002). Success varied by state. AM and AC had more than a 60% reduction, while AP reduced cases by 15% and RO, 9% (Massarani 2001).

FUNASA expanded the network of surveillance, diagnosis and treatment at the local level and therefore malaria cases were detected faster, leading to a more rapid break in the chain of transmission. The budget for disease control in the Amazon tripled to $54 million in 2001 leading Carlos Catão Prates Loiola to say: “we are seeing…an increase in the mobilisation and competence of state and local staff. But…the extra health ministry funding was critical.” (Massarani 2001). By 2002, diagnosis and accurate treatment had improved and it was possible to be properly diagnosed within 24 h in most of the affected areas. However, it was also argued that vector control was 20% of what it should have been (Loiola et al. 2002). In 2002 and 2003 there was another increase in malaria transmission (Marcano et al. 2004). In 2004, there were 350,000 cases of malaria in Brazil [455,448 cases (Brazilian Ministry of Health, unpublished observations)] (Silva-Nunes et al. 2006).

## Malaria control passes to the National Program for Malaria Prevention and Control (PNCM)

By 2005, malaria control had passed to the PNCM. Its objectives were to reduce mortality, severe malaria and overall malaria incidence (Coura et al. 2006). PNCM also encompassed a malaria information system (Malaria Epidemiological Surveillance Information System) for managing epidemiological data and consolidation of routine tasks like forwarding compiled results to regional offices and their headquarters. Monitored variables include city-level transmission estimates, drug dispensation and treatment failure. In 2007, 47 Amazonian municipalities accounted for 70% of malaria cases. By 2008, malaria had returned to numbers close to those seen in 1983 (314,420 cases) and the Amazonian API had dropped to 12.8. PNCM thought that their efforts to strengthen local capacity, diagnosis and treatment accounted for the reduction starting in 2006. In 2010, the Global Fund agreed to fund a Ministry of Health project aimed at strengthening local public health capacity in the Amazon by ensuring early diagnosis, timely treatment and extending bed net coverage, as well as municipal and state-level management. The artesunate mefloquine combination drug used in AC was being produced locally in adult and child doses, but in other Amazon states arthemeter plus lumefantrine was the drug of choice for *P. falciparum* cases. Due to the concentration of malaria cases in the Amazon, physicians outside of that area were less likely to consider diagnosing malaria when febrile patients presented (Oliveira-Ferreira et al. 2010).

Between 2010-2013, there has been a decline in malaria cases in Amazonian states of Brazil. Between 2012-2013, AM (-8%), MA (-12%), PA (-69%) and RO (-38%) all showed a decrease in malaria cases. AP (+1%), MT (-1%) and RR (0%) showed little to no change in the number of cases, while the number of cases in AC increased (28%). Among these states, reported malaria cases went from 246,608 in 2011 (unpublished governmental data reports 266,975 cases), to 227,379 in 2012 (unpublished governmental data report 243,752 cases), to 166,689 in 2013 (unpublished governmental data report 78,490 cases) (SIVEP-Malária 2014). The few cases that occurred outside of the Amazon Region were generally introduced to regions which were previously malaria endemic. However, a very few cases (0.05%) were due to autochthonous malaria along the southeastern Atlantic coastal forest (de Pina-Costa et al. 2014).

## History of drug use and first reports of resistance

*P. falciparum*’s resistance to quinine and CQ developed quickly in Brazil. Quinine was already the antimalarial of choice in Brazil at the beginning of the XX century. However, quinine resistance was first noted in 1907 in RJ and then again in 1910 after it was distributed as a prophylactic to railway workers in RO (Neiva 1910, da Silva & Benchimol 2014). CQ was first used from 1946-1947 in Belém and Santarém hospitals at a dosage of 1,500 mg. *P. falciparum* recrudescence was reported, leading one author to suggest that there were already CQ resistant parasites circulating prior to CQ’s introduction (Reyes 1981). It was distributed as chloroquinised salt in PA from 1952-1953, PR from 1952-1954, MA from 1953-1954, state of Minais Gerais starting in 1956, SC state starting in 1956, AP state starting in 1957 and along the Amazon River starting in 1959 (Payne 1988). This likely led to its use at less than adequate dosages by the general population and facilitated the development of resistance (de Souza 1992).

It has generally been established that South American CQ resistance began on the border of Colombia and Venezuela and then spread throughout the continent. This may be incorrect or only partially true. After CQ resistance was reported in Colombia in 1961 in an international journal, CQ resistance was suddenly reported in Porto Velho, Belém and along the 300 km Belém-Brasília Road. Malaria recrudescence after CQ treatment was reported in AP, AM and RR in the same year (Box et al. 1963, Reyes 1981, de Souza 1992). This falls into a simple narrative described above.

However, Dr R Brito and A Pinheiro reported that there was CQ resistance in RO in 1954 to Heath Secretary of the Federal Territory of Guaporé and later at a National Health meeting (de Souza 1992). Based on this information, resistance could have spread from the western Brazilian Amazon to the rest of the Brazilian Amazon and the border of Colombia and Venezuela. If this were the case, the prevailing understanding of how CQ resistance spread in South America might be misinformed due to underreporting.

Regardless, in response to CQ resistance, an investigative centre was created by PAHO/WHO at the Santa Teresa Psychiatric Hospital in Ribeirão Preto, SP. By 1965 CQ resistance was again reported in Belém, as well as Manaus. Over the next two years it was again reported in PA and RR, as well as in the state of Espírito Santo and MG. Overall, drug susceptibility studies suggested that 56% of cases were CQ resistant during the 1960s (PAHO 1984). CQ resistance was reported in MT by 1969, but CQ resistance was first reported in AC in 1980 (Reyes 1981). In 1981, 25% of parasites in MA were resistant to CQ (Silva et al. 1984).

By the end of the 1970s, CQ resistance had increased substantially (PAHO 1984). By the 1980s, CQ resistance was reported throughout Brazil (Reyes 1981). In Belém, patients treated with CQ, amodiaquine and sulphadoxine pyrimethamine had a cure rate of 10% and less than 20% in Goiânia, GO in 1987 (*P. falciparum* resistance to amodiaquine was also reported in the 1960s) (de Souza 1992). In AC, 73% of samples were resistant to amodiaquine and 84% were resistance to CQ in the same year (Kremsner et al. 1989). Another study conducted in 1997 with 10 patients showed that 100% of the samples were RI or RII resistant to CQ and amodiaquine, while one was RIII resistant to quinine (4 from RO, 4 from MT and 2 from PA) (Segurado et al. 1997). In 1998 in PA, only 4% of parasites were CQ sensitive in Macapá and Serra do Navio, where miners around the Serra do Navio had been using chloroquinised salt for prophylaxis. However, they were all susceptible to mefloquine, amodiaquine and quinine (Póvoa et al. 1998). According to molecular studies, CQ resistance was prevalent by the early 1980s and did not disappear over time (Vieira et al. 2001,^2004^, Cortese et al. 2002, Ferreira et al. 2008, Mehlotra et al. 2008, Gama et al. 2009).

Like quinine and CQ, pyrimethamine resistance appeared soon after its initial use. Pyrimethamine was used in neighbouring Venezuela in 1956 with early reports of drug resistance by 1959 (Gabaldón et al. 1959,Maberti 1960). Sulphadoxine pyrimethamine was first used in trials at the beginning of the 1960s in response to reports of CQ resistance and then applied in public health interventions in the 1970s (Walker & Lopez-Antunano 1968). Between 1965-1967, a study conducted in four Brazilian localities showed that 102 out of 104 infections were cured when treated with sulfadoxine-pyrimethamine (PAHO 1984). In 1968, 18 strains collected from Brazil, Colombia and Venezuela were used to inoculate patients in SP. Some strains were shown to be mildly resistant to pyrimethamine (collected in Belém and Boa Vista, RR) or moderately resistant (Machado River, R, El Pescado, Colombia, Igarapé Mirim, PA, Barcarena, PA, Puerto Ayacucho, Venezuela, and Cripori River, close to PA), with one strain reported to have RII resistance (collected in Goiânia). However, all were sensitive to pyrimethamine when used in combination with various sulphonamides. In the same study, three of fifteen “attacks” treated with quinine were not cured (Walker & Lopez-Antunano 1968).

Resistance to sulfadoxine in Brazil appears to have taken longer to develop than other drugs. Sulfadoxine-pyrimethamine resistance was first reported in 1972 in Brazil (de Souza 1992). A retrospective study of treatment between 1974 -1979 showed that in GO, five patients did not clear parasites from their blood with the application of sulfadoxine-pyrimethamine and another showed that 164 patients showed RII level resistance to sulfadoxine-pyrimethamine in Amazon. Sulfadoxine-pyrimethamine resistance was also reported in MA in 1978 (Alecrim et al. 1982,Alecrim 1986, de Souza 1992). In the early 1980s, sulfadoxine-pyrimethamine treatment failed 16-63% of the time in Brazil (Peterson et al. 1991). In the western Amazon, only 30% of cases treated with sulfadoxine-pyrimethamine were cured, though the cure rate was 75% in the eastern Amazon (de Souza 1992). During 1980 and 1981, RI sulfadoxine-pyrimethamine resistance was seen 25% of parasites in Paragominas, Brazil (WHO 1985). In 1981, 16% of parasites were resistant to sulfadoxine-pyrimethamine in MA (Silva et al. 1984). By 1982, RI resistance to sulfadoxine-pyrimethamine was reported in patients from the Tapajós River, PA, Maués, AM, Ariquemes, RO, and MT. RII resistance was reported in Maués and Humaita. Perhaps more troubling, RIII resistance was reported along BR-319, a road that connects Manaus to Porto Velho (Alecrim et al. 1982). By 1984, 30-50% of infections were no longer cured by sulfadoxine-pyrimethamine in Brazil (PAHO 1984), though it was as high as 60% in the eastern Amazon (de Souza 1992). In 1985, 52 patients infected with Amazon strains and treated with sulfadoxine-pyrimethamine showed RI in 32.7%, RII in 42.3% RII and RIII in 7.7% of cases (Alecrim 1986). In 1987, sulfadoxine-pyrimethamine resistance in eastern Amazon was 90% and 92% in western AC (Kremsner et al. 1989, de Souza 1992)

By the end of the 1980s, 90% of parasites were sulfadoxine-pyrimethamine resistant. Brazil shifted to quinine-tetracycline as the standard treatment for uncomplicated*P. falciparum*, followed by quinine and doxycycline and mefloquine plus primaquine as a secondary-line drug (Duarte et al. 1996, Gama et al. 2009). While there was an illegal market for mefloquine among miners, there was no reported resistance except for a few case that might have been due to patient malabsorption (Póvoa 1993). A report from 2001 showed that 23.8% of uncomplicated malaria cases in AC were resistant to quinine plus doxycycline (Leal et al. 2003). Parasites collected from MT showed reduced quinine sensitivity, though only a few showed high quinine resistance in the late 1990s. Parasites were also susceptible to mefloquine and halofantrine (Zalis et al. 1998). According to molecular studies, low-level sulphadoxine pyrimethamine resistance was widespread in 1987 and higher levels prevalent by the late 1990s (Peterson et al. 1991, Vasconcelos et al. 2000, Cortese et al. 2002, Gama et al. 2009).

More recently, new combination therapies have been used in Brazil. From 2006-2008, a pilot study of fixed-dose artesunate plus mefloquine (ASMQ) for the treatment of*P. falciparum* was applied at three sites in AC to good effect (Santelli et al. 2012). This result spurred the Ministry of Health to implement ASMQ nationally during 2007-2008, along with arthemeter plus lumefantrine. In rural locations in the Amazon, quinine plus antibiotics was still wildly used to treat *P. falciparum*. However, large hospitals in the Amazon used artemisinin plus lumefantrine (Camargo et al. 2009). Mefloquine was not recommended for use in endemic areas as it has a very long half-life and can generate *P. falciparum* resistant strains.

Unlike *P. falciparum*, *P. vivax* only recently showed signs of CQ resistance. Reports from 2000 and 2007 suggest that* P. vivax* is becoming resistant to CQ in Manaus. In a study of 109 volunteers with uncomplicated infection that completed the in vivo test in 2004-2005, 11 showed clinical failure when treated with 150 mg CQ tablets every 24 h for 27 days (Barbosa et al. 2007).

## Concluding remarks

The incidence and distribution of malaria across Brazil have varied over modern history. Despite this, once it reached the Amazon interior, it bloomed. Even now the Amazon Basin accounts for 99% of Brazil’s reported cases with regional increases in incidence often associated with large scale public works or economic shifts. Furthermore, as sporadic outbreaks and autochthones zones outside the Amazon attest, malaria can easily regain the ground it loses due to flagging surveillance and control. Man-made environmental changes like those inadvertently caused by dams, open pit mining or deforestation of the Amazon Basin are obvious self-inflicted wounds to successful malaria control. On the other hand, it appears that deforestation outside the Amazon Basin along the coastline in AP and in northern SP reduced the incidence of malaria by reducing the number of bromeliads and thus the number of *An. (Kerteszia) cruzi *(Gadelha 1994, Tauil 2011)*.*

The question of how to control or even eliminate malaria from the Brazilian Amazon has vexed regional experts for as long as Brazil has existed. The XX century showed that efficient antimalarials and insecticides are effective control methods, if they don’t fail. Unfortunately, they all seem to eventually fail, albeit hopefully at a much slower rate in the future due to the lessons learned during CEM.

During the rise of these chemicals to ascendency, there was an alternate school of thought which suggested that a more holistic approach to public health would engender the same benefits. As our review notes, the living conditions of poor Brazilians in rural locations have supported endemic malaria. However, improvements to domiciles and work environments are inherently more difficult than distributing antimalarials and applying insecticides when the communities most impacted are*ad hoc* collections of agriculturalists or miners where malaria is constantly reintroduced.

Yet Brazil’s past successes with such holistic methods suggest that they can reduce the incidence of malaria when consistently applied. The successful provision and use of bed nets is a simple way to improve the quality of housing when public health officials cannot patch the open air of an Amazonian home. More difficult than the distribution of bed nets is to confront and fill the ditches and other water collections near communities that may move with the next economic boom. Aside from public health efforts, increases in *per capita* income over the long term may indirectly reduce the incidence of malaria in the Amazon Basin through the improvement of basic living conditions by citizens themselves.

Perhaps the most obvious lesson from Brazil’s history is that malaria control needs to be placed on permanent and well-funded ground (da Silva & de Oliveira 2002). Another obvious lesson is that public works programs and efforts to settle the Amazon require careful public health surveillance and sanitary engineering prior to shifts in human populations. A more ambiguous lesson is that combining malaria control with other public health service may be ill-advised, based on past experiences in Brazil and Venezuela (Griffing et al. 2014).
